# In this month’s Bulletin

**DOI:** 10.2471/BLT.22.000122

**Published:** 2022-01-01

In the editorial section Akhil Bansal (2) argues for equitable distribution of vaccines to prevent COVID-19. Kristen Meagher et al. (3) examine gender and leadership in the health-sector response to conflict-affected settings.

Andrei Shukshin (6–7) reports on an initiative in the Russian Federation to improve food sources by the building of local greenhouses in remote communities. Rose Gana Fomban Leke talks to Gary Humphreys (8–9) about rethinking approaches to malaria elimination and her efforts to support the next generation of scientists in Cameroon.

## Afghanistan, Angola, Bangladesh, Benin, Bhutan, Burkina Faso, Burundi, Cambodia, Central African Republic, Chad, Comoros, Democratic Republic of the Congo, Djibouti, Eritrea, Ethiopia, Gambia, Guinea, Guinea-Bissau, Haiti, Kiribati, Lao People's Democratic Republic, Lesotho, Liberia, Madagascar, Malawi, Mali, Mauritania, Mozambique, Myanmar, Nepal, Niger, Rwanda, Sao Tome and Principe, Senegal, Sierra Leone, Solomon Islands, Somalia, South Sudan, Sudan, Timor-Leste, Togo, Tuvalu, Uganda, United Republic of Tanzania, Vanuatu, Yemen, Zambia.

### Measuring progress on health development goals

Luhua Zhao et al. (40–49) document data gaps.

## Afghanistan, Bangladesh, India, Maldives, Nepal, Pakistan, Sri Lanka

### Nutrition and health interventions 

Phuong Hong Nguyen et al. (20–29) find unequal coverage for women and children.

## African Union 

### Which antibiotic?

Jessica Craig et al. (50–59) compare national treatment guidelines.

## Bangladesh 

### Kangaroo mother care 

ANM Ehtesham Kabir et al. (10–19) study implementation barriers.

## Cameroon, Indonesia and Pakistan.

### Reaching every linguistic community

Pierpaolo Di Carlo et al. (78–80) provide information in minority languages.

## India 

### Hypertension screening 

Sanjay K Mohanty et al. (30–39) quantify missed opportunities.

## Mozambique 

### Maternal health services and hepatitis B virus

Anne Loarec et al. (60–69) pilot a mother-to-child transmission prevention programme.

## Global

### Sustainable health equity

Arachu Castro et al. (81–83) advocate for measures to address climate change and social inequalities.

### Reducing the footprint of food

Hannah Nissan et al. (70–77) describe climate-sensitive nutrition programmes.

